# Predicting nutrient excretion from dairy cows on smallholder farms in Indonesia using readily available farm data

**DOI:** 10.5713/ajas.20.0089

**Published:** 2020-05-12

**Authors:** Windi Al Zahra, Corina E. van Middelaar, Imke J.M de Boer, Simon J. Oosting

**Affiliations:** 1 Animal Production Systems Group, Wageningen University & Research, P.O. Box 338, 6700 AH, Wageningen, The Netherlands; 2 Department of Animal Production and Technology, Faculty of Animal Science, IPB University (Bogor Agricultural University), Bogor, West Java 16680, Indonesia

**Keywords:** Dairy Cows, N and P Excretion, Smallholder Dairy Farms

## Abstract

**Objective:**

This study was conducted to provide models to accurately predict nitrogen (N) and phosphorus (P) excretion of dairy cows on smallholder farms in Indonesia based on readily available farm data.

**Methods:**

The generic model in this study is based on the principles of the Lucas equation, describing the relation between dry matter intake (DMI) and faecal N excretion to predict the quantity of faecal N (Q_FN_). Excretion of urinary N and faecal P were calculated based on National Research Council recommendations for dairy cows. A farm survey was conducted to collect input parameters for the models. The data set was used to calibrate the model to predict Q_FN_ for the specific case. The model was validated by comparing the predicted quantity of faecal N with the actual quantity of faecal N (Q_FNACT_) based on measurements, and the calibrated model was compared to the Lucas equation. The models were used to predict N and P excretion of all 144 dairy cows in the data set.

**Results:**

Our estimate of true N digestibility equalled the standard value of 92% in the original Lucas equation, whereas our estimate of metabolic faecal N was −0.60 g/100 g DMI, with the standard value being −0.61 g/100 g DMI. Results of the model validation showed that the R^2^ was 0.63, the MAE was 15 g/animal/d (17% from Q_FNACT_), and the RMSE was 20 g/animal/d (22% from Q_FNACT_). We predicted that the total N excretion of dairy cows in Indonesia was on average 197 g/animal/d, whereas P excretion was on average 56 g/animal/d.

**Conclusion:**

The proposed models can be used with reasonable accuracy to predict N and P excretion of dairy cattle on smallholder farms in Indonesia, which can contribute to improving manure management and reduce environmental issues related to nutrient losses.

## INTRODUCTION

The number of dairy cattle in Indonesia has increased from 503,000 in 2014 to 550,000 in 2018 due to an increase in the demand for milk and a governmental decision to support the growth of the national dairy sector [1]. This increase in dairy cattle, mainly kept on smallholder dairy farms, has enhanced the negative consequences associated with the lack of manure management on those farms, resulting in large amounts of discharged manure. Because dairy production in Indonesia is concentrated in regional clusters, this discharge of manure leads to high concentrations of nitrogen (N) and phosphorus (P) in local rivers and groundwater of densely populated areas, impacting human health and natural ecosystems [2]. Manure management on smallholder dairy farms must be improved to reduce those negative consequences.

Approximately 84% of the smallholder dairy farms in Indonesia discharge at least part of their manure into the environment [3]. While the urinary or liquid fraction is totally discharged, part of the solid fraction may be collected and sold to manure traders, crop or flower farmers, or used on the farm itself, i.e., as organic fertiliser or to produce biogas for cooking. In some cases, the solid manure fraction is composted before being sold or applied as fertiliser.

To improve manure management, information about N and P excretion of dairy cattle is needed. This information can be used to estimate nutrient losses from different manure treatment options and to quantify differences in nutrient use efficiency among farms and manure management systems. To accurately predict related environmental problems and losses of N and P, the N and P excretion in faeces and urine should be calculated separately. This separation between faecal and urinary fraction is important, because these fractions are differently managed at the Indonesian dairy farms. Moreover, the nature of losses differs between both manure fractions: ammonia volatilization is much higher for the urinary than for the faecal fraction [4].

Quantifying N and P excretion from dairy cattle can be done by different methods, including actual measurements (e.g. in feeding trials) or by means of mathematical modelling. Both methods have advantages and drawbacks. Feeding trials are generally used to analyse the digestibility of individual feed ingredients and complete diets, providing an accurate estimate of N and P excretion [5]. This approach, however, is laborious, expensive, and difficult to scale to the level of a dairy farm. Mathematical modelling offers a method to predict N and P excretion using on-farm data, including animal and dietary characteristics [6–8]. A linear regression equation with dry matter intake (DMI) and crude protein (CP) intake was used to predict the N excretion of Chinese Holstein dairy cows [6]. Similarly, a linear function of DMI and P intake (PI) was used to estimate P excretion in high productivity lactating Holstein dairy cows [8].

Mathematical models may be useful to predict N and P excretion on dairy farms, but many of these models are developed based on input-output relationships that are applicable only to the specific condition under which the input-output model was assessed. Hence, such models may not be suitable for the Indonesian situation, because of differences not just regarding dietary composition and animal productivity, but also regarding, among others, environmental factors, breed, and production level which can have a substantial effect on the relation between feed intake and N and P excretion [9]. So far, a generic model to predict N and P excretion of dairy cows on smallholder farms in Indonesia is not available. Therefore, this study aims to provide models to accurately predict N and P excretion of dairy cows on smallholder farms in Indonesia based on readily available farm data. In this study, we calibrated and evaluated a generic model to predict faecal N excretion and we subsequently applied this model in combination with existing guidelines to predict N and P excretion in faeces and urine for 144 cows on 30 smallholder dairy farms in Indonesia.

## MATERIALS AND METHODS

The generic model in this study is based on the principles of the Lucas equation, describing the relation between DMI and faecal N excretion [10–13]. In addition, we used the guidelines of the National Research Council (NRC) to calculate the daily N and P requirements of dairy cows [14], which were then used to calculate the excretion of urinary N and faecal P. The following section describes the model and guidelines. Subsequently, a description of the collection of farm data is provided, followed by a description of the calibration and evaluation of the faecal N model. Finally, we illustrate the reliability of the models by presenting the effective sample sizes required to identify a difference between treatments.

### Modelling N excretion

The Lucas equation describes the apparent digestibility of nutrients, independent of the feed, based on true digestibility, and the endogenous loss of that nutrient in the faeces, (equation 1a) and is widely used in nutrient digestibility studies for ruminants, but most for protein and N [10–13]. The general Lucas equation for N is:

(eq.1a) DN=m TN+b

where DN is the concentration of digestible nitrogen in ingested dry matter (g/100 g), TN is the concentration of total nitrogen in ingested dry matter (g/100 g), the slope (m) is the true digestibility of the protein in the feed (fraction) and the intercept (b) is the concentration of endogenous N in ingested dry matter (g/100 g). If we multiply the left and right-hand-side of equation (1a) with DMI (g/animal/d), we get equation 1b.

(eq.1b) DNI=(m×TNI)+(b×DMI)

where DNI is the digestible N intake (g/animal/d), and TNI is the total N intake (g/animal/d).

This reformulated Lucas equation enables prediction of the quantity of N in faeces (Q_FN_) (g/animal/d) since Q_FN_ is the difference between total N intake (TNI) and digestible N intake (DNI), equation 2a:

(eq.2a) $${\text{Q}}_{\text{FN}}=\text{TNI}-\text{DNI}$$

Subsequently, we substitute DNI in equation (2a) by the reformulated Lucas equation (1b), yielding our equation to predict the quantity of faecal N given in equation (2b or 2c):

(eq.2b) $${\text{Q}}_{\text{FN}}=\text{TNI}-[(\text{m}\times \text{TNI})+(\text{b}\times \text{DMI})]$$

(eq.2c) $${\text{Q}}_{\text{FN}}=[(1-\text{m})\times \text{TNI}]-(\text{b}\times \text{DMI})$$

The quantity of urinary N (Q_UN_) (g/animal/d) can subsequently be calculated by subtracting total N retained (N_Ret_) (g/animal/d) for producing milk, pregnancy, growth and scruf protein, and Q_FN_ (g/animal/d) from the total N intake (TNI) (g/animal/d), given in equation 3.

(eq.3) $${\text{Q}}_{\text{UN}}=\text{TNI}-{\text{N}}_{\text{Ret}}-{\text{Q}}_{\text{FN}}$$

Subsequently, the quantity of total N in manure (Q_TN_) (g/animal/d) is calculated as the sum of Q_FN_ (g/animal/d) and Q_UN_ (g/animal/d), given in equation 4.

(eq.4) $${\text{Q}}_{\text{TN}}={\text{Q}}_{\text{FN}}+{\text{Q}}_{\text{UN}}$$

The N_Ret_ (g/animal/d) can be calculated for lactating, dry cows and young cows based on the NRC guidelines [14]. The scurf protein consists of protein loss from skin, skin secretions, and hair, and is calculated as 0.3×BW^0.60^ (live weight). The retained N for milk production equals N in milk (N_Milk_) (g/animal/d) and is calculated by multiplying the daily milk production (g/animal/d) with the protein concentration of milk, divided by 6.38 which is the conversion factor from milk protein to N. The retained N for foetal growth in a pregnant animal (N_Preg_; g/animal/d) is calculated by dividing the metabolizable protein requirement for pregnancy (MP_Preg_) by 6.25. For cows between 190 to 279 days of pregnancy, MP_Preg_ is computed as:

(eq.5) $${\text{MP}}_{\text{Preg}}=[(0.69\times \text{days in pregnancy})-69.2\times (\text{CBW}/45)]/{\text{Eff}}_{\text{MPPreg}}$$

where, CBW is calf birth weight (kg), and Eff_MPPreg_ is the efficiency of use of metabolised protein (MP) for pregnancy, which is assumed to be 0.33.

For our model we assume that N retained for growth (N_Growth_) of lactating and dry cows is zero. In young cows, N_Growth_ (g/animal/d) is estimated by dividing the metabolizable protein for growth (MP_Growth_) by 6.25. The MP_Growth_ is computed based on equation 6:

(eq.6) $${\text{MP}}_{\text{Growth}}={\text{NP}}_{\text{g}}/(0.834-(\text{EQSBW}\times 0.00114)$$

where NP_g_ is net protein for gain and is calculated from SWG× (268−[29.4×(RE/SWG)]). SWG is the shrunk weight gain and is assumed to equal 13.9×NE_Growthdiet_^0.9116^×EQSBW^−0.6837^. NE_Growthdiet_ is the net energy requirement for growth available (Mcal/d) and calculated as (0.84 BW^0.355^×WG^1.2^)×0.69. BW is the current live weight of an animal (kg) and WG is the weight gain per animal (g/d). EQSBW is the equivalent shrunk body weight and is calculated as SBW×(478/MSBW). SBW is shrunk body weight (animal weight after an overnight fast without feed or water) and being set at 96% of the current live weight. MSBW is the mature shrunk body weight and being set at 96% of the expected mature live weight (MW). The retained NE (RE) (Mcal/d) is assumed to equal 0.0635×EQEBW^0.75^×EQEBG^1.097^. EQEBW is equivalent empty body weight (weight without ingesta), and assumed to equal 0.891× EQSBW. EQEBG is the equivalent empty body weight gain, being calculated as 0.956×SWG.

### Modelling P excretion

Unlike N that is in faeces and urine, P is mainly in faeces. The P that is contained in urine of dairy cows is minimal and, therefore, can be neglected [14–16]. The daily quantity of P excreted via faeces (Q_FP_; g/animal/d) is calculated as the differences between daily PI (g/animal/d) and P retained (P_Ret_; g/animal/d) for milk production, pregnancy, and growth per day (equation 7). To calculate PI (g/animal/d), information about DMI (g/animal/d) and P concentration of the ingested DM (g/kg) is required (equation 8).

(eq.7) $${\text{Q}}_{\text{FP}}=\text{PI}-{\text{P}}_{\text{Ret}}$$

(eq.8) PI=DMI×P concentration of ingested DM

The retained P for milk production equals P in milk (P_Milk_; g/animal/d) and is calculated by multiplying the daily milk production (kg/animal/d) with the P concentration of milk (g/kg). P retention for pregnancy (P_Preg_; g/animal/d) is calculated for cows in 190 to 279 days pregnancy based on equation 9:

(eq.9) $${\text{P}}_{\text{Preg}}=0.02743{\text{e}}^{(0.05527-0.000075\text{t})\;\text{t}}-0.02743{\text{e}}^{\left(0.05527-0.000075(\text{t}-1))\;(\text{t}-1\right)}$$

where t is day of gestation.

The retained P for growth (P_Growth_) of lactating and dry dairy cows is assumed to be zero. In young cows, P retention for growth (P_Growth_; g/animal/d) is estimated based on equation 10:

(eq.10) $${\text{P}}_{\text{Growth}}=[1.2+(4.635\times {\text{MW}}^{0.22})\times ({\text{BW}}^{-0.22})]\times (\text{WG}/0.96)$$

where the MW is the estimated expected mature live weight per animal (kg), BW is current live weight per animal (kg), and WG is the weight gain per animal (g/d).

### Data collection

A farm survey was conducted to collect data for model calibration to predict N and P excretion of dairy cows in Indonesian smallholder farms. The survey was conducted in December 2017 in the Lembang district, West Java, Indonesia. This district is known as one of the largest clusters of smallholder dairy farms in Indonesia. We selected 30 out of the 300 dairy farms which participated in a baseline survey conducted within the project Sustainable Intensification Dairy Production in Indonesia [3]. The district has approximately 5,000 dairy farms. The selection of the 30 farms was purposively done to include four distinct manure management systems. However, the difference in manure management systems is not relevant for this paper, and, therefore, will not be discussed here. All farmers were members of a dairy cooperative in Lembang, West Java.

The input parameters to calibrate and evaluate the models to predict N and P excretion were the animal’s diet and production stage including herd composition (lactating, dry, and young cows), daily milk yield, manure composition and the live weight of the animals (Table 1). The number of days in pregnancy for dry cows was provided by the farmers during the interview (range from 210 to 240 days). The live weight (BW) of each cow was estimated based on the hearth girth using the Schoorl equation [19]. Information about calf birth weight (CBW), expected mature live weight (MW) and weight gain (WG) was not available from the survey and, therefore, was estimated based on literature representing the Indonesian situation. CBW per animal was assumed to be 40 kg [20], MW per animal was assumed as 500 kg, and WG per animal was assumed to equal 450 g/d [21].

The feed for the animals was offered three times daily (i.e., in the morning, at noon and in the afternoon) and the quantity of offered feed (g/animal/d) was measured at each feeding time using a weighing scale. The net individual diet on fresh weight basis (g/animal/d) was determined based on the difference between feed offered and feed left-over with the latter being collected the day after before the first feeding time. The feed leftover comprised the roughages only. At each farm we collected feed samples of all feeds offered such as roughages, compound feed, and agro-industrial by-products. Dry matter (DM), ash, CP, and P concentration of each feed product of each farm were measured in the laboratory.

During the farm survey, from each lactating cow we measured daily milk production (g/animal/d) using a weighing scale and we collected a milk sample twice a day during milking time (morning and afternoon). Each milk sample was analysed for N and P concentration (g/kg). Furthermore, a sample of fresh faeces was collected from each farm for analysis of DM, N, and P concentration (g/kg).

The laboratory analysis of DM concentration of the feed samples was determined by drying at 105°C until constant weight and ash was determined by ashing at 600°C. We assumed that the nutritional composition of feeds was similar for offered feed and feed left-overs. The DM concentration of the fresh faeces was determined in a 105°C drying process. The N analysis was done by using the standard Kjeldahl method. The N value was multiplied by 6.25 for feed and faeces, and by 6.38 for milk to determine the protein concentration. The P concentration was analysed using a titrimetric method for the feed sample and a microcolorimetric method for the milk and faeces sample. The laboratory analysis of feed, milk, and faeces was conducted in the Faculty of Animal Science, IPB University, Indonesia.

### Model calibration and evaluation

The farm data were used to calibrate and evaluate the Q_FN_ model. To calibrate the Q_FN_ model for the Indonesian context, the data set was divided into a training data set (3/5 of the total data set) and a testing data set (the remaining 2/5). The training data set was used to estimate the intercept and the slope of equation (1a) (Table 2). The testing data set was used for model evaluation. The training and testing data were randomly selected.

As the first step of model evaluation, we predicted the quantity of N in the faeces (Q_FNPRED_; g/animal/d) using equation (2c). Following this, we compared the values of Q_FNPRED_ with the actual measurement of faecal N from the independent data set (Q_FNACT_; g/animal/d). The Q_FNACT_ values were calculated by multiplying the values of indigestible DMI (IDMI; g/animal/d) (Table 2) with the N concentration in faeces (g/kg) that was obtained from the laboratory analysis (Table 1). Finally, the proposed Q_FNPRED_ model was statistically evaluated against the Q_FNACT_ by using the mean average error (MAE) in equation [11] and the root mean square error (RMSE) in equation [12]. Both RMSE and MAE were presented as absolute and as relative value. The mean square error (MSE) consists of the bias error, the slope error, and the random error [22]. A low score of MAE and RMSE indicates a better model performance.

(eq.11) $$\text{MAE}=\frac{1}{n}\sum_{i=1}^{n}\left(X_{observation}-X_{prediction}\right)$$

(eq.12) $$\text{RMSE}=\sqrt{\frac{1}{n}\sum_{i=1}^{n}{\left(X_{observation}-X_{prediction}\right)}^{2}}$$

In addition, the predicted intercept and slope of Q_FN_ model for smallholder dairy farms (Q_FNPRED_) were compared to the intercept and slope reported for the Lucas equation for N in literature [12]. The literature values for intercept and slope of the Lucas equation for N are 92% and −0.61 g N/100 g DMI, respectively.

### Effective sample size

The accuracy of a model determines the effective sample size (i.e. the number of dairy cows required) in a study to detect a specific difference between two treatments (e.g., before and after an intervention) [23]. A larger sample size is needed when a less accurate model is used. The accuracy of a model is expressed by the reliability score which is equal to the coefficient of determination (R^2^) of the model. In this study, the R^2^ was the R^2^ from the regression of Q_FNPRED_ on Q_FNACT_. The R^2^ from the actual measurement of faecal N (Q_FNACT_) was assumed as without error (R^2^ = 1). The Cohen method [23] was used to determine the effective sample size for Q_FNPRED_ and Q_FNACT_ (equation 13):

(eq.13) $$n=\frac{2\delta^{2}}{{\left(d\right)}^{2}}$$

where, *n* is the effective sample size and *δ* is the critical value of *t*, and the *t* is the critical *t*-value in the t-test distribution given as *t_1−α_* and *t_1−β_*. The δ is calculated as δ =(*t_1−α_*−*t_1−β_*). The *α* indicates the probability of a type I error and *β* the probability of a type II error. The *d* is the standardized effect size and calculated as (*m_A_*-*m_B_*/*σ*) where *m_A_* and *m_B_* are the means of populations A and B, respectively (e.g. with and without an intervention), and *σ* is the population standard deviation. The two populations (A and B) were assumed to have equal variances and an equal reliability coefficient, *α* was set at p = 0.05 (one-tailed), and *β* at p = 0.20. In this study, we calculated the effective sample sizes in order to detect a specific difference of Q_FN_ ranging from 1 to 30 g/animal/d. All statistical analyses in the present study were performed in R (R Core Team, 2018).

## RESULTS

### Farm survey findings

The 30 smallholder dairy farmers in this study kept a total of 144 dairy cows, i.e. 106 lactating cows, 12 dry cows, and 26 young cows. The young cows counted 12 replacement females with an average age between 6 to 24 months, and 14 calves (males and females) with an average age between 4 and 5 months. Lactating cows had an average live weight of 433 kg, and an average milk yield of 13 kg per day. Dry cows had an average live weight of 419 kg and were 210 to 240 days in pregnancy. Young cows had an average live weight of 278 kg. Table 3 provides an overview of the feed types and the average feed intake per animal class. There was no difference between the type of feed fed to lactating cows, dry cows, and young cows. Overall, on a DM basis, the diet of lactating cows, dry cows and young calves, but at different intake levels, consisted of roughages such as elephant grass, road side grass, and rice straw (48%), agro-industrial by-products, such as tofu waste and cassava waste (22%), and concentrates (28%). Relatively low amounts of other feed products such as legumes (0.3%), premix (0.01%), banana stalks (0.09%), and crop leftovers (0.6%) were fed. These products were excluded from the model since the amount was insignificant, and the usage was inconsistent across farms.

Table 4 shows the average nutrient composition of feed, milk and faeces. The average CP concentration of 140 g/kg DM in concentrate feed was at the lower range of CP levels in concentrates for dairy cattle generally used in Indonesia (140 to 210 g/kg DM) [24]. The average protein concentration of 34 g/kg for milk met the minimum Indonesian requirement of 27 g/kg milk [25]. The average N concentration of 24 g/kg DM for the faeces was within the range of 22 to 26 g/kg DM as found in literature [26,27] and the P concentration of 7 g/kg DM for the faeces was in the range of 5.2 to 7.4 g/kg DM as found in literature [28].

Table 5 presents the feed intake per animal class. Results show that the quantity of feed differed among animal classes. Intake of DM, N, and P were higher in lactating cows than in dry cows, which in turn had higher intake of these nutrients than young stock. On average, lactating cows consumed 22% more than dry cows, and 46% more than young cows. Similarly, on average, the NI was 25% higher in lactating cows compared to dry cows and 48% higher compared to young cows. The average PI was 27% higher in lactating dairy cows compared to dry cows and 48% higher compared to young cows.

### Model calibration and evaluation

The training data set (n = 86) was used to estimate the intercept and the slope for equation (1a). The intercept was found to be −0.60 g/100 g DMI and the slope was found to be 0.92. This implies that the amount of metabolic faecal N increases by 0.6 g per 100 g DMI with a predicted true digestibility of the protein in the feed of 92%. The proposed Q_FN_ model for Indonesian smallholder dairy farms is therefore:

(eq.14) $${\text{Q}}_{\text{FN}}\;(\text{g}/\text{animal}/\text{d})=[0.08\times \text{TNI }(\text{g}/\text{animal}/\text{d})+0.60\times \text{DMI }(100\;\text{g}/\text{animal}/\text{d})]$$

**Figure 1:**
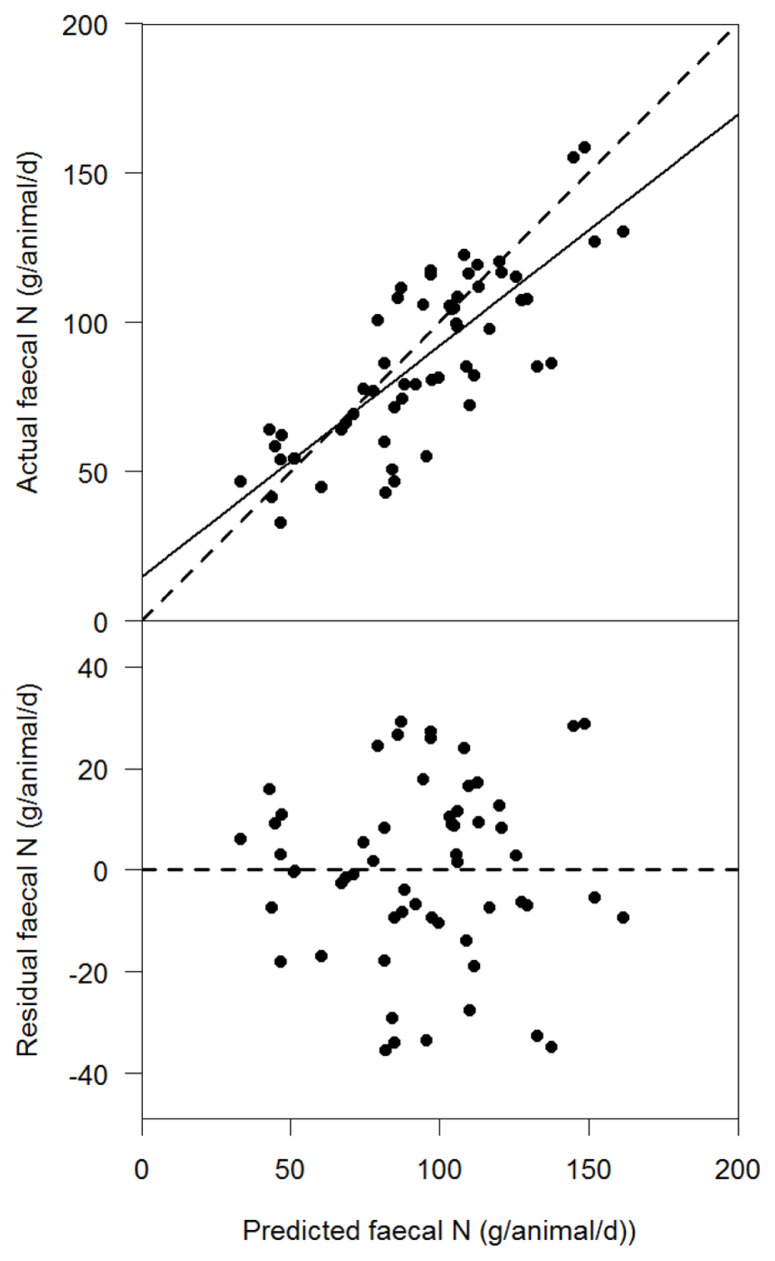
Plot of model evaluation of Q_FN_ for the data set. The solid line indicates the regression line of prediction faecal N (Q_FNPRED_) and actual faecal N (Q_FNACT_). The dashed line is the line of unity. Q_FN_, quantity of faecal N; Q_FNPRED_, predicted quantity of faecal N; Q_FNACT_, actual quantity of faecal N.

The testing data set (n = 58) was subsequently used to evaluate the Q_FN_ model in equation 14, by comparing Q_FNPRED_ with Q_FNACT_ (Figure 1). The coefficient of determination (R^2^) of Q_FNPRED_ and Q_FNACT_ was 0.63 (residual standard error = 17.6, p<0.05). In this regression line, the intercept was significantly different from zero (p = 0.0003), however, the slope did not significantly differ from one (p = 0.16). The MAE was 15 g/animal/d which translates to 17% deviation of Q_FNPRED_ from the Q_FNACT_. The RMSE was 20 g/animal/d which translates to 22% deviation of Q_FNPRED_ from the Q_FNACT_. The bias error of the MSE was 9%, the slope error was 12% and the random error was 79%. The slope and intercept which we estimated for equation 2c were similar to those reported in literature [12].

### Effective sample size

**Figure 2:**
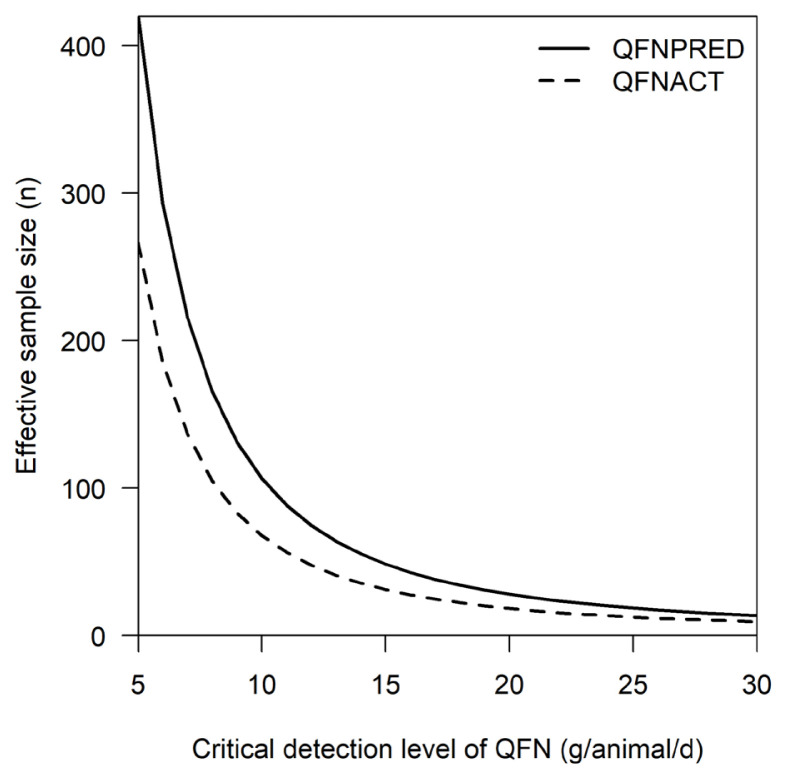
The relationship between effective sample size of dairy cows (n) and a specific difference of Q_FN_ (g/animal/d) in two alternative models (Q_FNPRED_, R^2^ = 0.63 and Q_FNACT_, R^2^ = 1). The solid line indicates the Q_FNPRED_ and the dashed line indicates the Q_FNACT_. Q_FN_, quantity of faecal N; Q_FNPRED_, predicted quantity of faecal N; Q_FNACT_, actual quantity of faecal N.

The effective sample size i.e. the number of dairy cows required in an experimental treatment to detect a specific difference between Q_FN_ of different treatments was compared between Q_FNPRED_ (i.e., derived from equation (14)) and Q_FNACT_ (i.e., derived from measurements). The relationship between effective sample size of dairy cows (n) and a specific difference of Q_FN_ (g/animal/d) in two alternative models (Q_FNPRED_; R^2^ = 0.63 and Q_FNACT_; R^2^ = 1) is illustrated in Figure 2. To detect a specific difference in Q_FN_ of 10 g/animal/d, for example, requires 68 animals when using Q_FNACT_, while 107 animals are needed when using Q_FNPRED_. For specific differences higher than 20 g/animal/d the effective sample size did not differ much between the two models.

### Model application

Equation (14) and the NRC guidelines [14] were used to predict N and P excretion and retention for all dairy cows in the data set (n = 144). Table 6 shows the average prediction of N and P excreted and retained (g/animal/d) per animal class. The average Q_FN_ was higher for lactating cows (107 g/animal/d, 38% of TNI) than for dry cows (83 g/animal/d, 39% of TNI) and young cows (57 g/animal/d, 39% of TNI). Similarly, the average Q_UN_ was higher for lactating cows (111 g/animal/d, 40% of TNI), than for dry cows (99 g/animal/d, 47% of TNI) and young cows (60 g/animal/d, 41% of TNI). Overall, the average Q_FN_ was 96 g/animal/d and Q_UN_ was 101 g/animal/d. The average N_Ret_ was 63 g/animal/d for lactating cows (22% of TNI), 29 g/animal/d for dry cows (14% of TNI), and 30 g/animal/d for young cows (20% of TNI). In the case of Indonesian smallholder dairy farms, on average 22% of TNI was retained and the remaining 78% of TNI was found in manure, with 38% in the faeces and 40% in the urine.

The average Q_FP_ was 63 g/animal/d (89% of PI) for lactating cows, 47 g/animal/d (90% of PI) for dry cows, and 32 g/animal/d (86% of PI) for young cows. The average P_Ret_ was 8 g/animal/d (11% of PI) for lactating cows, 5 g/animal/d (10% of PI) for dry cows, and 5 g/animal/d (14% of PI) for young cows. In the case of Indonesian smallholder dairy farms, on average 12% of PI was retained and 88% of PI was found in the manure. Average daily N and P excretion per farm (three lactating, one dry and one young cow) is approximately 947 g N and 268 g P.

## DISCUSSION

Since it is very difficult to sample manure and assess manure quantity at dairy farms we calibrated and evaluated the Q_FN_ model, and subsequently predicted Q_FN_, Q_UN_, and Q_FP_ in our case region based on feed intake and composition, milk production and its composition, and manure composition. The Lucas equation is an important element of the Q_FN_ model, and the model calibration for dairy cattle at the farms in the study area was essentially an evaluation of the Lucas equation for the Indonesian situation. Our estimate of true N digestibility equalled the standard value of 92% in the original Lucas equation, whereas our estimate of metabolic faecal N was −0.60 g/100 g DMI, with the standard value being −0.61 g/100 g DMI. Our estimates of true N digestibility and metabolic faecal N, furthermore, were similar to those reported in literature [12]. Hence, the standard Lucas equation for N seems to apply under a wide array of conditions, including Indonesian smallholder dairy farms [29]. Consequently, the Q_FN_ model presented in this study can be applied under very different circumstances, and the standard values from the Lucas equation can likely be used.

To test the robustness of model, we applied a calibration/evaluation approach instead of using a sensitivity analysis. Results of the model evaluation showed that the Q_FNPRED_ model had a relatively high relative MAE (17%) and relative RMSE (22%). In literature [30] errors of 20% were found during the quantification of potential and feed-limited growth of three beef cattle breeds by a generic model which was followed by a model evaluation on independent experimental data. This error is comparable to our findings. The systematic errors (bias error and slope error) were limited and the major source of error was the random error (79%). The relatively high error could in part be attributed to the fact that some model parameters such as DDM had to be derived from literature [17,18]. The specified information of DDM for many feed types, for example the roughage, is limited for the Indonesian situation, whereas the variation in DDM quality of roughage among farmers is expected to be high. In addition, the Q_FNACT_ that was used as actual value for model evaluation and for the estimation of the effective sample size was considered without error. In reality, the Q_FNACT_ also has an estimation error because of errors related to sampling, to laboratory analysis and to the DDM values used to estimate Q_FNACT_. Hence, the MAE and RMSE of Q_FNPRED_ when evaluated against a real direct assessment (full collection of faecal and urinary excretion separately and compositional analysis of each fraction) will likely be higher than when compared to the Q_FNACT_ in the present study.

We used the NRC guideline to estimate the nutrient requirements. In Indonesia, it is widely used because of the absence of a national system to estimate dairy cattle feed requirements. Nevertheless, since the cattle were high grade Holstein Friesian cows, we believe that most NRC predictions are applicable to the breed in Indonesia, and because the weather conditions in the research area are relatively mild, they also apply to the climatic conditions.

We selected the farms randomly and we collected feed samples from each farm, so we assume the farm and feed samples represented the actual situation. The variation in composition of agro-industrial by-products and concentrate was low with limited difference between dry and rainy season because they were produced by agro-industries which use standardized processes, hence delivering standard quality, even of the by-products they sell. In addition, the concentrate was produced by the dairy cooperative with the aim to deliver standardized quality to the members of the cooperative. The roughage differed only slightly between seasons [31]. Since the Lembang area is small, conditions for all farmers are similar. Hence, variation in composition between diets and within feeds was small in the Lembang area.

The average predicted Q_FN_ was lower (96 g/animal/d) than some values reported in literature (147 to 242 g/animal/d) [5,6,8]. The difference between our estimate and these reported values could be due to the lower DMI and NI in our study. To verify this conclusion, we inserted the DMI and NI values from literature [5,6,8] into our Q_FN_ model, and the result showed that the relative deviation of predicted Q_FN_ values from the values reported in previous studies varied from −15% to 19%.

We calculated nutrient use inefficiency for nitrogen (NUI_N_) by expressing excreted N as percentage of NI. In our study, this NUI_N_ was 78% meaning that 78% of N intake ended up in manure, and only 22% in milk and meat. The NUI_N_ in literature [5,8,32] was lower than the one found by us i.e. 70% to 72%. This could mean two things: either N losses via manure were higher from the cattle in our study caused by a low efficiency of N utilization in the animal which could be caused by limitation by other nutrients, by the genetic potential of the animals or by health-related factors [30] or it could just be that too much N was offered through the diets. These reasons imply that improving feeding management for example through nutritionally balanced rations [33], adjustment of the dairy genetics to the production potential at the present feed base and animal health care may potentially reduce nutrient excretion.

Some mathematic models to predict N and P excretion of dairy cows are developed based on input-output relations from dairy farms in a specific context. Although such models are compelling because they only require limited data to predict the N and P excretion, they may fail when applied in systems different from the one for which they were created [34]. Applying such existing models to the case of smallholder dairy farms in Indonesia, therefore, may lead to over or under estimation of N and P excretion because of differences in feed input (lower feed intake) and animal characteristics (lower milk production and body weight). Therefore, a generic model is proposed. The generic model in this study described the process of N digestion and N utilisation for maintenance, growth and production based on well-established methods generally applied in animal nutrition (Lucas equation and NRC). Additionally, this generic model is calibrated and evaluated, and the model evaluation showed that the model can be used to estimate faecal N at smallholder dairy farms in Indonesia.

## CONCLUSION

We developed, calibrated and evaluated a generic model to predict Q_FN_ from dairy cattle on smallholder farms in Indonesia using readily available farm data, and applied this model, in combination with existing guidelines of the National Research Council, to predict N and P excretion in faeces and urine for 144 dairy cows on 30 farms. In conclusion, the proposed models can be used with reasonable accuracy to predict N and P excretion of dairy cattle on smallholder farms in Indonesia using readily available farm data. The model can be used as a basic tool to improve manure management and to reduce nutrient losses in Indonesian smallholder dairy farms.

## Figures and Tables

**Table 1:** Input parameters to calibrate and evaluate the models to predict nitrogen (N) and phosphorus (P) excretion on smallholder dairy farms

Input parameters	Data required	Method
Feed intake	Type of feed	Interview with the farmers
	Daily feed intake in fresh weight basis per animal class	On-farm measurement
	Concentration of:	Laboratory analysis1)
	Dry matter	
	Ash	
	Crude protein	
	P	
	Daily feed intake on dry matter basis per animal class	Daily feed intake in fresh weight basis × DM concentration of diet
	Concentration of digestible dry matter	Literature [17,18]
Feed requirement	Nitrogen for producing milk (N_Milk_)	[14]
	Nitrogen for pregnancy (N_Preg_)	
	Nitrogen for growth (N_Growth_)	
	Phosphorus for producing milk (P_Milk_)	[14]
	Phosphorus for pregnancy (P_Preg_)	
	Phosphorus for growth (P_Growth_)	
Milk	Daily milk yield	On-farm measurement
	N concentration of milk	Laboratory analysis1)
	P concentration of milk	
Manure	Concentration of	Laboratory analysis1)
	Dry matter	
	N	
	P	
BW2)	Heart girth of the animal	On-farm measurement

1) Laboratory analysis was conducted at Faculty of Animal Science IPB University, Indonesia.

2) Live weight.

**Table 2:** Parameters and equations to calibrate and evaluate the Q_FN_ model

Parameters	Equations
DMI (g/animal/d)	The net daily feed intake in fresh weight basis (g/animal/d) × DM concentration (g/kg)
DDMI (g/animal/d)	DMI (g/animal/d) × DDM concentration (g/kg)
IDMI (g/animal/d)	DMI (g/animal/d) – DDMI (g/animal/d)
TNI (g/animal/d)	DMI (g/animal/d) × CP concentration in feed/6.25 (g/kg)
IDNI or Q_FNACT_ (g/animal/d)	IDMI (g/animal/d) × N concentration in the faeces (g/kg)
DNI (g/animal/d)	NI (g/animal/d) – IDN (g/animal/d)

Q_FN_, quantity of faecal N; DMI, dry matter intake; DM, dry matter; DDM, digestible dry matter; DDMI, digestible dry matter intake; IDMI, indigestible dry matter intake; TNI, total nitrogen intake; CP, crude protein; IDNI, indigestible nitrogen intake; Q_FNACT_, actual quantity of faecal N; DNI, digestible nitrogen intake; NI, nitrogen intake; IDN, indigestible nitrogen intake.

**Table 3:** An overview of the feed types and the average of feed intake (mean±standard error) by lactating, dry and young cows on a dry matter basis (g/animal/d) on 30 smallholder dairy farms in the Lembang, West Java, Indonesia

Feed type	Lactating cows	Dry cows	Young cows
Elephant grass	3,620±284	4,319±1,130	3,310±744
Road side grass	1,342±293	752±656	571±396
Rice straw	949±137	515±276	485±251
Cassava waste	1,230±151	713±253	295±159
Tofu waste	1,944±211	1,881±496	1,049±258
Concentrate	4,796±351	2,590±940	1,763±453
Total	13,881±632	10,769±603	7,472±466

**Table 4:** Average nutrient composition of feed, milk, and faeces samples collected (mean±standard error)

Feed type	n1)	DM	CP	P	Ash	DDM
Nutrients composition of feed (g/kg DM)						
Elephant grass	27	178±11	101±6	4±0.1	112±6	529 [17]
Road side	9	188±15	103±7	5±0.4	101±9	489 [18]
Rice straw	11	319±32	90±3	3±0.3	198±13	408 [17]
Tofu waste	15	155±7	201±2	3±0.2	33±2	865 [18]
Cassava waste	17	181±13	61±5	4±0.5	28±10	768 [17]
Concentrate	30	876±3	140±1	7±0.4	73±3	861 [17]
	
	**n**1)		**Protein**		**P**	
	
Nutrient composition of milk (g/kg)	106		34±0.4		0.6±0.005	
	
	**n**1)	**DM**	**N total**		**P**	
	
Nutrient composition of faeces (g/kg DM)	30	138±10	24±0.5		7.0±0.2	

DM, dry matter; CP, crude protein; P, phosphorus; DDM, dry matter digestibility; N, nitrogen.

1) Number of sample.

**Table 5:** Feed intake on a dry matter basis per animal class (g/animal/d) used to calibrate and evaluate N and P excretion model

Parameters	Minimum	Maximum	Mean±SE
Lactating dairy cows (n = 106)			
DMI	6,548	22,048	13,881±632
DDMI	4,706	16,159	9,738±232
IDMI	1,815	7,370	4,142±114
CPI	859	3,214	1,756±49
NI	138	514	281±8
IDN	42	273	101±4
DN	67	319	180±5
PI	26	141	71±3
Dry dairy cows (n = 12)			
DMI	5,615	19,476	10,769±603
DDMI	4,611	13,215	7,300±700
IDMI	833	6,261	3,500±500
CPI	798	2,166	1,320±111
NI	128	346	211±18
IDN	24	130	84±8
DN	43	228	127±16
PI	26	125	52±8
Young dairy cows (n = 26)			
DMI	3,403	15,548	7,472±466
DDMI	2,624	10,400	4,853±398
IDMI	589	5,147	2,578±198
CPI	654	1,886	918±69
NI	51	302	147±11
IDN	38	144	63±6
DN	32	190	84±8
PI	15	102	37±4

SE, standard error; DMI, dry matter intake; DDMI, digestible dry matter intake; IDMI, indigestible dry matter intake; CPI, crude protein intake; NI, nitrogen intake; IDN, indigestible nitrogen intake; DN, digestible nitrogen intake; PI, phosphorous intake.

**Table 6:** Predicted N and P excreted and retained (mean±SE) in lactating cows, dry cows and young cows on 30 smallholder dairy farmers in Lembang, West Java, Indonesia

Parameters estimate	Lactating cows	Dry cows	Young cows	Average
Q_FN_	107±2.5	83±8.2	57±4.3	96±2.6
Q_UN_	111±5.3	99±11.9	60±6.0	101±4.5
Q_TN_	218±7.8	182±20.1	117±10.3	197±7.1
N_Ret_	63±1.9	29±0.02	30±2.3	54±1.80
Q_FP_	63±5.6	47±8.4	32±4.2	56±2.5
P_Ret_	8±0.1	5±0.1	5±0.05	7±0.2

SE, standard error; Q_FN_, quantity of faecal N; Q_UN_, quantity of urinary N; Q_TN_, quantity of total N; N_Ret_, retained N; QFP, quantity of faecal P; P_Ret_, retained P.
